# The effect of continuity of care on the incidence of end-stage renal disease in patients with newly detected type 2 diabetic nephropathy: a retrospective cohort study

**DOI:** 10.1186/s12882-018-0932-3

**Published:** 2018-06-05

**Authors:** Yun Jung Jang, Yoon Soo Choy, Chung Mo Nam, Ki Tae Moon, Eun-Cheol Park

**Affiliations:** 1 The Health Insurance Dispute Mediation Committee, Ministry of Health & Welfare, Sejong Government Complex, Sejong City, Republic of Korea; 2 Department of Health Policy and Management, Graduate School of Public Health, Yonsei University, Seoul, Republic of Korea; 3 Department of Public Health, Graduate School, Yonsei University, Seoul, Republic of Korea; 4 Institute of Health Services Research, Yonsei University, Seoul, Republic of Korea; 5 Department of Preventive Medicine & Institute of Health Services Research, Yonsei University College of Medicine, 50 Yonsei-ro, Seodaemun-gu, Seoul, 120-752 Republic of Korea; 6 National Evidence-Based Healthcare Collaborating Agency, Seoul, Republic of Korea

**Keywords:** Diabetic renal complication, Continuity of care, ESRD, Disparities in health outcomes

## Abstract

**Background:**

Diabetic nephropathy requires periodic monitoring, dietary modification, and early intervention to prevent the disease severity within limited resource settings. To emphasize the importance of continuous care for chronic diseases, various studies have focused on the association between continuity of care (COC) and common adverse outcomes. However, studies aimed at understanding the effect of COC on the incidence of chronic diseases, such as end-stage renal disease (ESRD), are few. The aim of this study was to determine whether there is an association between COC and the incidence of ESRD among patients with diabetic nephropathy. Moreover, we identified individual- and hospital-level factors associated with the incidence of ESRD among diabetic nephropathy patients.

**Methods:**

We conducted a retrospective cohort study using the administrative National Health Insurance claims data from 2005 to 2012 in the Republic of Korea. The dependent variable, a binary variable, was the incidence of ESRD due to diabetic renal complication. In addition, using the COC index as a binary variable with a cutoff point of 0.75, we divided patients into a ‘Good COC group’ (COC index≥0.75) and a ‘Bad COC group’ (COC index< 0.75). The survival analysis was performed using the Cox proportional hazards models.

**Results:**

Among 3565 diabetic renal complication patients, ESRD occurred among 83 diabetes mellitus patients (2.3%). Nephropathy patients with lower COC level (< 0.75) had 1.99 times higher risk of ESRD incidence (95% confidence interval [CI]:1.27–3.12). In addition, the lowest income level patients had higher hazard ratio (HR) of ESRD than the highest income level patients (HR: 1.69 95% CI: 0.95–2.98), while patients with disabilities had 2.70 higher HR of ESRD than patients without disabilities (95% CI: 0.64–43).

**Conclusions:**

Among patients with diabetic renal complication, higher continuity of care was associated with lower risk of ESRD. It is therefore recommended that continuous follow-up be encouraged to prevent ESRD among diabetic renal complication patients. Moreover, disparities in health outcomes between socially vulnerable groups including patients with disabilities and those in the lowest income level should be addressed.

**Electronic supplementary material:**

The online version of this article (10.1186/s12882-018-0932-3) contains supplementary material, which is available to authorized users.

## Background

Nephropathy causes approximately 48.0% of end-stage renal disease (ESRD) cases, and accounts for an annual medical cost of more than 1 billion dollars in the United States [1]. The major complications of nephropathy, which dramatically increase medical costs in the Republic of Korea, include kidney transplants, dialysis, percutaneous transluminal coronary angioplasty, coronary artery bypass surgery, and leg amputation, according to the stage of diabetic retinopathy and nephropathy [2]. Furthermore, in Korea, the number of patients with diabetes increased by 24.6% from 2010 to 2015, and among them, approximately 5.8% were diagnosed with diabetic renal complication [3, 4]. These diabetic complications need periodic monitoring, dietary modification, and early intervention. Therefore, to effectively manage chronic diseases such as diabetes within limited resource settings, policymakers have developed clinical practice guidelines, with attention focused on the benefits of continuity of care (COC) [5–7].

COC is an essential concept for high-quality patient care, and it entails how patients’ experiences are linked with care over time or the connectedness of the discrete elements of care [8, 9]. Furthermore, COC has positive effects on various outcomes; for example, it encourages patient satisfaction, treatment adherence [10, 11], or increases the recognition rate of diabetes, and leads to better glycemic control among diabetic patients [12–16]. Moreover, higher COC with the usual provider for diabetes mellitus is associated with a lower risk of future or preventable hospitalizations for long-term diabetic complications, and it might further decrease medical costs [17, 18]. Therefore, COC and management of the physician-patient relationship should be reinforced in the early stages of the disease condition for a more effective management of diabetes [19].

Higher provider COC is characterized by better approaches to the sharing of disease information, without information asymmetry, while eliminating conflicts of interest between patient and practitioners [20].

Despite evidence that COC is associated with better patient outcomes, empirical studies of associations between COC and health outcomes in the Korean population are rare. Moreover, few studies exist on the effect of COC on the incidence of other chronic diseases like ESRD. Therefore, the aim of this study was to investigate the association between continuity of ambulatory care and the incidence of chronic kidney disease among patients with renal diabetic complications, using the administrative claims data from the Republic of Korea’s National Health Insurance (NHI) scheme. Moreover, we identified individual- and hospital-level factors associated with the incidence of ESRD among diabetic nephropathy patients.

## Methods

### Data

A retrospective cohort study was conducted using population-based data collated from the Korean NHI database from 2005 to 2013. Data was obtained from the Korean National Health Insurance Service-National Sample Cohort (NHIS-NSC) claims database for 2002–2012, which includes information on approximately 1 million Koreans since 2002. The NHIS-NSC used a 2.5% (*n* = 1,025,340) stratified random sampling method, with the goal of providing representative, useful health insurance and health examination data to public health researchers and policy makers. Of note, additional data handling to account for missing data is unnecessary, as the data was already processed by the NHIS upon researcher request.

Based on the NHI data, newly diagnosed diabetic renal complication (International Classification of Diseases tenth edition diagnosis code (ICD-10): E11.2) patients were identified. The data were also analyzed to determine the association between COC level and the incidence of ESRD. The data were stratified according to age, sex, region, health insurance type, disability status, residence area, income decile, insulin treatment, and the severity of complications. The data included participant characteristics as well as medical institution variables (hospital category, the number of beds, the number of doctors, and hospital location) as covariates. All individual- and hospital-level characteristics were measured at baseline in 2005. The diagnoses were based on the ICD-10 codes.

### Participants

**Fig. 1:**
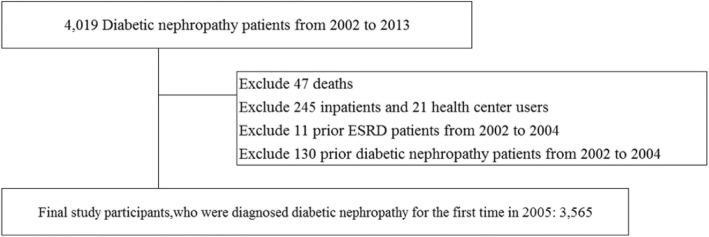
Flow diagram of study participants

The total number of individuals with diabetic nephropathy (ICD-10: E11.2) was 4019 from 2002 to 2013. Of these, 3706 patients were diagnosed in 2005, and to specifically analyze newly diagnosed diabetic nephropathy patients, we excluded 130 prior diabetic nephropathy patients and 11 prior ESRD patients from 2002 to 2004, and included the first diabetic nephropathy (ICD-10:E11.2) patient from 2005. Because we focused on the continuity of ambulatory care for 8 years among surviving diabetic nephropathy patients, and observed the incidence of ESRD from 2005 to 2013, we excluded 47 patients who died during this period. Moreover, in calculating COC index, we limited included patients to those on hospital outpatient visits due to diabetic nephropathy; hence, 245 inpatients and 21 health center users were excluded. Using these criteria, the final study sample included 3565 diabetic nephropathy patients. The flow diagram of the study participants’ selection is shown in Fig. 1. Furthermore, to perform survival analysis using a Cox proportional hazards models, we chose an appropriate follow-up period. In patients diagnosed with ESRD, we set the follow-up period as the time from the start date to the date of ESRD occurrence. On the other hand, we set the end date as the death date or the end of 2013 for the non-ESRD group and deceased patients.

### Independent variable: COC

COC was included as an independent variable, and included both prescription continuity and the clinical management of diseases. Moreover, the measurement of COC considered the characteristics of the Korean medial delivery system, where patients are allowed to freely choose their preferred primary care. Therefore, the COC index was chosen to measure consistency of care.

According to the findings of previous studies, COC can be determined in many ways, including the use of the Modified Continuity Index, and the Most Frequent Provider Continuity [21–25].

As devised by Bice et al. (1977), the COC index can be calculated by the total patient contact times with the medical service providers and the number of healthcare providers. The COC index ranges from 0 (when patients’ visits to medical institutions occur with different providers at each visit) to 1 (when patients present to the same medical institution for outpatient services on many occasions). Therefore, an index close to 1 implies that the patient frequently utilizes a particular medical service provider; hence, the COC level is high.

The formula for determining the COC index is shown below:

$$ COC=\frac{\sum \limits_{j=1}^M{n}_j^2-N}{N\left(N-1\right)} $$

Where N = the total number of visits, M = the number of available medical service providers, and n_j_ = the number of visits to the j^th^ providers.

From previous studies [26, 27], we used the cutoff point of 0.75 for the COC index, whereby patients were classified as having good COC if they had 75% of their ambulatory visits with the same practitioner over the study period. On the other hand, patients who made less than 75% of visits to the same doctor were classified as having bad COC. This measure has been validated extensively in previous studies. Therefore, we used the COC index because the caregivers are not determined in advance in Korea, and we calculated patients’ visits until the occurrence of ESRD. Furthermore, the number of days from the first diagnosis to ESRD occurrence was calculated. The COC index was categorized as a binary variable into the ‘Good COC group’ (COC index≥0.75), when diabetic patients most often visited the same medical institutions for the treatment of diabetic complications, and the ‘Bad COC group’ (COC index< 0.75), when diabetic patients visited different medical institutions on most occasions, similar to the findings of previous studies [28, 29].

### Dependent variable: The incidence of ESRD

In this study, the dependent variable was the incidence of ESRD. To measure this, we identified patients who developed ESRD using ICD-10 codes such as hemodialysis (O7020, O9991), peritoneal dialysis (O7062), or kidney transplant (R3280), among newly diagnosed patients with diabetic renal complications from 2005 to 2012.

### Covariates

We examined covariates that affected the occurrence of ESRD and the COC of patients with diabetic renal complications. Furthermore, we included both individual- and hospital-level characteristics as covariates. Individual-level characteristics adjusted for included sex, age, residence area, health insurance type, income level, insulin treatment, disability status, and disease severity. Age was categorized into five groups (under 50, 50–59, 60–69, 70–79, and over 80 years). Residence area was divided into three groups (metropolitan, urban, and rural areas). Health insurance type was categorized as health insurer and Medicaid user. Furthermore, we divided income into four quartiles of 25% each, from the lowest (Q1) to the highest (Q4).

Disease severity was measured by the Korean Diagnosis-Related Group (KDRG) codes and comorbidities. Related with diabetes mellitus were six-digit KDRG codes including K60000, K60001, K60002, and K60003. Among the numbers, the last digits (0, 1, 2, and 3) represent the Patient Clinical Complexity Level (PCCL). By definition, ‘0’ implies without severe status or comorbidity, ‘1’ indicates the presence of accompanying mid-level complications or comorbidities, ‘2’ refers to the presence of more severe complications or comorbidities, while ‘3’ implies the most severe complications or comorbidities. The increasing severity implies that the larger the number, the higher the severity of the disease. Furthermore, PCCL was defined as the severity value according to the number of complications related to diabetes mellitus using the KDRG codes.

Moreover, we adjusted for both hospital- and individual-level characteristics. We included hospital classifications (general hospital, hospital, and clinic), categorized according to the Korean medical law. Based on hospital organization type, acute care hospitals were included in ‘hospitals’, while ‘general hospitals’ were defined by The Korean Hospital Association as hospitals with 80 or more beds and at least eight major clinical departments. Such clinical departments include internal medicine, general surgery, pediatrics, obstetrics and gynecology, radiology, emergency medicine, and pathology. Moreover, number of beds, number of doctors, and hospital location (metropolitan, urban, rural) were included as covariates. The capital and largest city (“Seoul”) was included in “Metropolitan area,” while “Urban” was defined as cities classified by administrative districts (such as Busan, Daegu, Incheon, Gwangju, Daejeon, and Ulsan). For both the number of beds and the number of doctors, the medians were reported; all of these covariates and all diagnostic information were collected at baseline (2005).

### Statistical analysis: Survival analysis

For this study, we used SAS version 9.4 (SAS institute Inc. Cary NC, USA) statistical software and the significance level was set at 5%. First, the distribution of demographic characteristics among diabetic patients with renal complications were assessed at baseline. Baseline categorical variables were expressed as numbers and percentages and were compared using the *χ*^2^ test.

Second, survival analysis was performed to determine the effect of COC on the incidence of ESRD among diabetic patients with renal complications using the Cox proportional hazards models. Furthermore, the Kaplan-Meier curve was plotted to demonstrate the essential assumption of proportional hazard regression model. Moreover, we calculated the mean time to the diagnosis of ESRD by each categorical variable using the log-rank test. The adjusted hazard ratios (HRs) for the incidence of ESRD by the Cox proportional hazards models were calculated using the PROC PHREG procedure in SAS. Third, after adjusting for other individual- and hospital-level characteristics on the regression model, we conducted subgroup analysis to determine the association between COC and the incidence of ESRD by residence area, income level, and disability to determine the effect of socio-economic status on patients’ COC.

## Results

### Demographic characteristics of the study population

A total of 3565 patients with the diagnosis of non-insulin-dependent diabetes mellitus with renal complications were included in this retrospective cohort study from 2005 to 2012. Table 1 presents the demographic characteristics of the study population at baseline (2005). To calculate the COC index, we defined the time period as the time from when a diabetic nephropathy patient started using hospital ambulatory care to the event time, whether ESRD was diagnosed or not, by the end of follow-up. During the 8-year follow-up from 2005 to 2012, 83 patients were diagnosed with ESRD with a mean ± standard deviation (SD) COC level of 0.398 ± 0.492. Among the 3482 patients with no ESRD, the mean ± SD COC level was 0.612 ± 0.487.

**Table 1:** Distribution of subject characteristics by ESRD occurrence at baseline 2005

	Total	ESRD occurrence						Person-year	Incidence Rate (×10^−5^)	*p*-value
		Yes			No					
Patient level										
Sex										
Male	1937	44		(2.3)	1893		(97.7)	8597.9	511.8	0.81
Female	1628	39		(2.4)	1589		(97.6)	7350.2	530.6	
Age (years)										
Under 50	738	9		(1.2)	729		(95.0)	3625.3	248.3	0.16
50–59	926	24		(2.6)	902		(94.1)	4169.8	575.6	
60–69	1097	26		(2.4)	1071		(93.4)	4958.0	524.4	
70–79	666	20		(3.0)	646		(93.2)	2665.8	750.2	
80 and more	138	4		(2.9)	134		(94.2)	529.1	755.9	
Residence area										
Metropolitan	829	18		(2.2)	811		(97.8)	3849.9	467.5	0.52
Urban	883	17		(1.9)	866		(98.1)	4105.7	414.1	
Rural	1853	48		(2.6)	1805		(97.4)	7992.4	600.6	
Health insurance type										
Health insurance	3471	80		(2.3)	3391		(97.7)	15,694.6	509.7	0.59
Medical aid	94	3		(3.2)	91		(96.8)	253.5	1183.2	
Income										
Q1 (Low)	816	27		(3.3)	789		(96.7)	3360.8	803.4	0.16
Q2	747	12		(1.6)	735		(98.4)	3361.4	357.0	
Q3	787	18		(2.3)	769		(97.7)	3694.2	487.2	
Q4 (High)	1215	26		(2.1)	1189		(97.9)	5531.7	470.0	
Insulin treatment										
Yes	160	3		(1.9)	157		(98.1)	15,312.6	522.4	0.69
No	3405	80		(2.4)	3325		(97.7)	635.5	472.1	
Disabled type										
Yes	482	28		(5.8)	454		(94.2)	1919.1	1459.0	<.0001
No	3083	55		(1.8)	3028		(98.2)	14,028.9	392.0	
PCCL index^b^										
0	3469	80		(2.4)	3389		(97.6)	15,722.7	521.5	0.23
1	61	2		(3.3)	59		(96.7)	131.5	1521.4	
≥2	35	1		(4.2)	34		(97.1)	93.9	1064.9	
Continuity of care^a^										
Good (0.75 ≤ COC index)	2164	33		(1.5)	2131		(98.5)	9926.7	332.4	<.0001
Bad (COC index< 0.75)	1401	50		(3.6)	1351		(96.4)	6021.4	830.4	
Hospital level										
Hospital classification										
General hospital	2040	52		(3.5)	1968		(96.5)	9266.5	777.0	<.0001
Hospital	97	11		(11.0)	86		(89.0)	408.1	245.1	
Clinic	1428	20		(1.4)	1408		(98.6)	6273.5	159.4	
Number of beds		787.5	±	461.0	475.7	±	508.6			<.0001
Number of doctors		220.2	±	164.3	136.2	±	194.0			<.0001
Location										
Metropolitan	973	19		(2.0)	954		(7.5)	4608.6	412.3	0.63
Urban	1016	24		(2.4)	992		(6.5)	4681.2	512.7	
Rural	1576	40		(2.5)	1536		(5.3)	6658.3	600.8	
Survey Year										
2005	587	15		(2.5)	572		(97.5)	4975.1	301.5	0.36
2006	440	5		(1.1)	435		(98.9)	2797.6	178.7	
2007	493	10		(2.0)	483		(98.0)	2611.7	382.9	
2008	526	12		(2.3)	514		(97.7)	2278.4	526.7	
2009	544	17		(3.1)	527		(96.9)	1797.4	945.8	
2010	457	14		(3.1)	443		(96.9)	1073.1	1304.6	
2011	255	8		(3.1)	247		(96.9)	343.7	2327.4	
2012	263	2		(3.8)	261		(96.2)	71.0	2817.8	
Total	3565	83		(2.3)	3482		(97.7)			

^a^COC index ranges from 0 to 1; 1 means that one patient has visited only one physician, and we could not put other continuity indices in this model at the same time because of multicollinearity among indices; Good is defined as COC score greater than 0.75, and Bad is defined as COC score less than 0.75

^b^The larger value was used regardless of whether the number of complications was related to diabetes mellitus or PCCL (Patient Clinical Complexity Level) using the KDRG code

Until 2012, among the 83 of 3565 diabetic nephropathy patients who developed ESRD, the overall incidence of ESRD was 520.4 per 100,000 person-years (95% confidence interval (CI) 419.7–645.4 per 100,000 person-years), and 332.4 and 830.4 per 100,000 person-years for the Good and Bad COC, respectively. The comparison of subject’s baseline characteristics showed that ‘Bad COC’ had higher incidence of ESRD than Good COC.

### Survival analysis for the incidence of ESRD using Kaplan-Meier curve

**Fig. 2:**
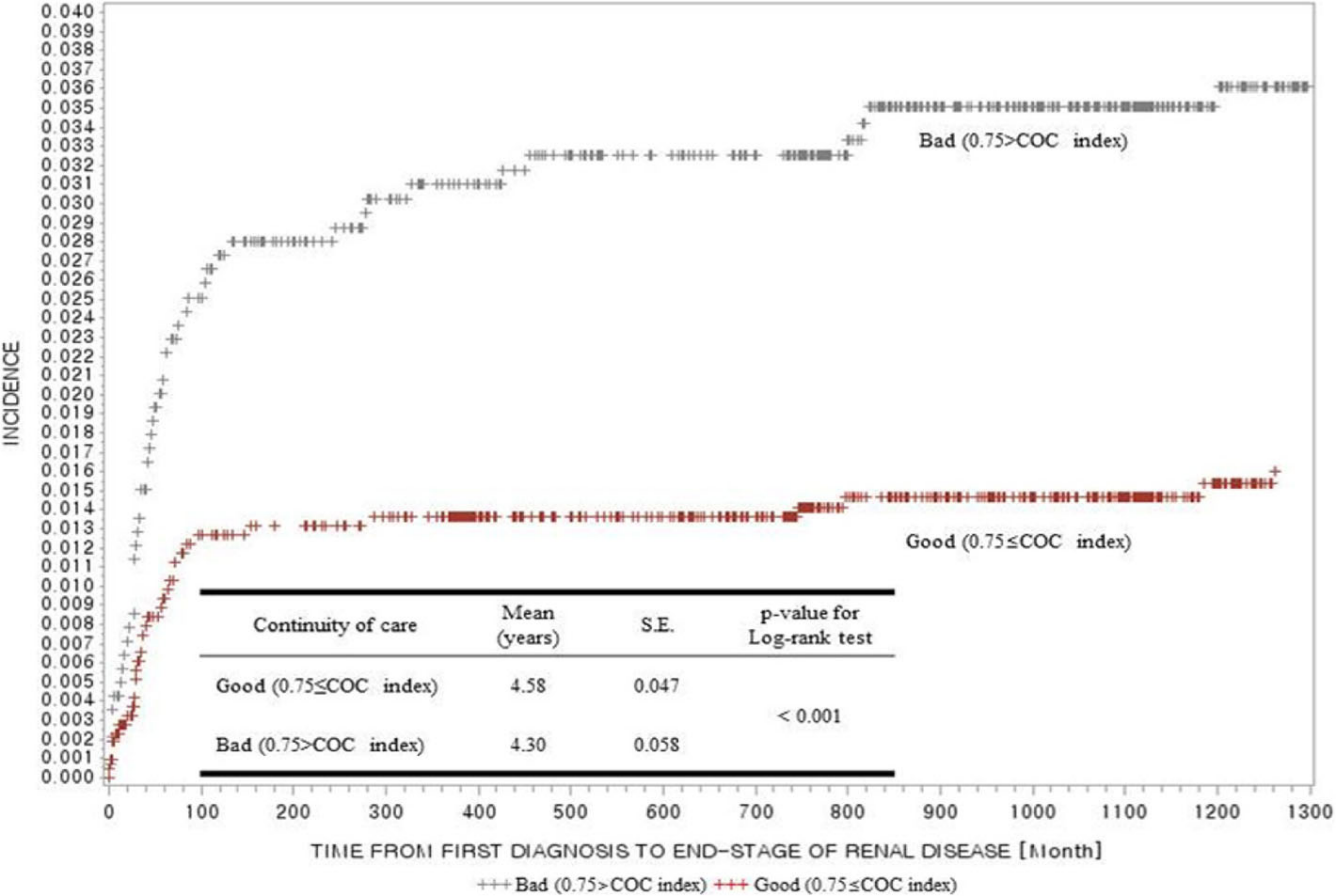
the Kaplan-Meier curve of the incidence probability of ESRD by divided COC groups and legend for exposure categories outcome with *p*-value for log-rank test

Fig. 2 shows the Kaplan-Meier curve of the incidence probability of ESRD by independent variables among the ‘Good’ and ‘Bad’ COC groups. According to the curve, those in the ‘Good COC group’ had lower incidence probability than those in the ‘Bad COC group’, and, with time, the incidence probabilities in both groups became sustained. Moreover, the median time of survival for individuals with Good and Bad COC were 4.58 and 4.30 years, respectively, in the Kaplan-Meier analysis.

### Cox proportional hazard model showing association between continuity of care and the incidence of end-stage renal disease

Table 2 shows the results of the Cox proportional hazards models showing the association between COC and the incidence of ESRD among diabetic patients with renal complications. After adjusting for all covariates (individual- and hospital-level characteristics), nephropathy patients with Bad COC had 1.99 times higher risk of ESRD incidence (95% CI: 1.27–3.12). We also implemented sensitivity analysis, which included 47 study participants who had died, and the results were similar to those in Table 2 (see Additional file 1: Table S1).

**Table 2:** Association between continuity of care and ESRD incidence*

	ESRD incidence				
	Hazard Ratio	95% CI			
Continuity of care					
Good (COC index≥0.75)	1.00	–		–	
Bad (COC index< 0.75)	1.99	(1.27	–	3.12)	

*Adjusted for sex, age, residence area, health insurance type, disability type, insulin treatment, *PCCL* index, hospital classification, number of beds, number of doctors, and hospital location

In Additional file 1: Table S1, factors associated with ESRD incidence are shown. Among individual-level characteristics, some variables were associated with ESRD incidence. The female sex showed a hazard ratio (HR) of 1.06, which was higher than that of males, while patients above 50 had higher probability of ESRD than those under 50. Patients who lived in rural (HR: 0.84 95% CI: 0.41–1.70), or urban areas (HR: 0.50 95% CI: 0.19–1.34) had lower incidence probability of ESRD than metropolitan citizens. In addition, those in the lowest income group had higher probability of developing renal failure than patients in the highest income group (HR: 1.69 95% CI: 0.95–2.98). However, none of these findings were statistically significant. Similarly, although patients with disabilities had higher ESRD incidence (2.70) than patients without disabilities, no significant difference (95% CI: 0.64–43) was found.

Among hospital-level characteristics, patients who utilized hospitals with either the highest number of beds or doctors had the highest probability of ESRD. Furthermore, when the hospital was located in a rural or urban area, the probability was also higher; however, these findings were not statistically significant. In addition, primary care level attendance showed a lower hazard (0.48) of ESRD than with general hospital attendance, with no statistically significant association (95% CI: 0.20–1.14).

### Subgroup analysis: Association between COC and the incidence of ESRD by residence area, income level, and disability

In Table 3, the results of the subgroup analysis of the association between continuity of care with and occurrence of ESRD occurrence by residence area, income, disability type, sex, and health insurance type are shown. After building the model adjusted for other individual- and hospital-level characteristics such as sex, age, health insurance type, hospital categories, number of doctors, number of beds, and hospital location, we conducted subgroup analysis to determine the association between COC and the incidence of ESRD by residence area, income level, disability type, sex, and health insurance type (Table 3).

**Table 3:** Subgroup analysis for the association continuity of care with occurrence of secondary diabetic complication by patient-level and hospital-level factors

	Continuity of care					*p*-value
	Good (0.75 ≤ COC index)	Bad (COC index < 0.75)				
	Hazard ratio (Ref.)	Hazard ratio	95% CI			
ESRD incidence						
Patient-level						
Sex						
Male	1.00	2.04	(1.09	–	3.82)	0.03
Female	1.00	1.86	(0.96	–	3.60)	0.07
Age						
Under 50	1.00	3.00	(0.75	–	11.98)	0.12
50–59	1.00	3.83	(0.36	–	1.95)	0.83
60–69	1.00	4.24	(1.78	–	10.09)	0.00
70–79	1.00	4.39	(1.59	–	12.07)	0.00
80 and more	1.00	1.41	(0.20	–	10.01)	0.73
Residence area						
Metropolitan	1.00	2.11	(1.04	–	5.30)	0.04
Urban	1.00	1.70	(0.62	–	4.65)	0.24
Rural	1.00	1.92	(1.06	–	3.48)	0.04
Health insurance type						
Health insurance	1.00	1.91	(1.21	–	3.03)	0.01
Medical aid	1.00	1.93	(0.17	–	21.57)	0.59
Income						
Q1 (Low)	1.00	2.04	(0.92	–	4.52)	0.08
Q2	1.00	1.70	(1.38	–	4.82)	0.02
Q3	1.00	0.94	(0.36	–	2.45)	0.91
Q4 (High)	1.00	2.27	(1.01	–	5.14)	0.05
Disabled type						
Yes	1.00	2.08	(0.96	–	4.48)	0.06
No	1.00	2.23	(1.43	–	3.49)	0.00
Hospital-level						
Hospital classification						
General hospital	1.00	1.86	(1.15	–	2.99)	0.01
Hospital	1.00	2.11	(0.64	–	6.91)	0.22
Clinic	1.00	2.73	(0.79	–	9.42)	0.11
Number of beds						
Q1 (Low)	1.00	2.60	(1.03	–	6.59)	0.04
Q2	1.00	1.39	(0.54	–	3.60)	0.50
Q3 (High)	1.00	2.00	(1.11	–	3.61)	0.02
Number of doctors						
Q1 (Low)	1.00	1.99	(0.72	–	5.48)	0.18
Q2	1.00	1.87	(0.67	–	5.25)	0.24
Q3 (High)	1.00	1.86	(1.06	–	3.27)	0.03
Location						
Metropolitan	1.00	1.96	(0.79	–	4.86)	0.15
Urban	1.00	3.46	(1.52	–	7.91)	0.00
Rural	1.00	2.05	(1.09	–	3.85)	0.03

Among patients with renal diabetic complications living in metropolitan areas, those with ‘Bad’ COC index (< 0.75) had 2.11 times higher probability of developing ESRD (95% CI: 1.04–5.30), and this was similar to the finding among patients living in rural areas (HR: 1.92, 95% CI: 1.06–3.48) or urban areas. However, in urban areas, no significant association was observed between COC and the incidence of ESRD.

Furthermore, among patients with diabetic complication in the lowest (Q1), second lowest (Q2), and the highest income levels, hospital medical services were more poorly utilized, the incidence probability was increased, and these associations were statistically significant. Moreover, patients with disabilities in the ‘Good’ COC group had a lower probability of ESRD compared with the ‘Bad’ COC group, with 1.86 higher incidence probability of ESRD (95% CI: 1.07–3.26).

Among hospital factors, hospitals in rural areas had a lower probability of ESRD for the ‘Good’ COC group than the ‘Bad’ COC group, with 2.05 higher incidence probability of ESRD (95% CI: 1.09–3.85). In addition, hospital classification was associated with the relationship between COC and the occurrence of ESRD. However, no significant association was observed between COC and the incidence of ESRD in hospitals and clinics.

## Discussion

According to findings of a previous study, COC consists of various elements not only the service provider and patient relationship, but also continuous data accessibility or total care management, which meant coherent delivery of care from different doctors [30]. Although COC is a complex concept, the core component is the consistency of contacts between specific patients and the providers [31]. Moreover, according to previous studies, the higher the severity or number of diabetic complications, the higher the probability of adverse patient outcomes, such as mortality and hospitalizations; therefore, there is need for consistency in the care of diabetic patients [32, 33]. In addition, newly diagnosed patients with hypertension, diabetes, hyperlipidemia, and those with bad COC levels, have higher risks of heart attack and cardiovascular heart diseases than patients with good COC levels [34].

The results of this study demonstrate that good COC in patients with diabetic renal complications is significantly associated with a lower probability of ESRD. Subgroup analysis was performed to determine whether there is an association between COC and ESRD by residence area, income level, and disability, revealing a higher probability of ESRD among patients with bad COC index (< 0.75) who lived in metropolitan and rural areas. Moreover, diabetic patients (who either had disabilities or were in the lowest income level) with bad COC level had higher probability of ESRD, but there was no statistically significant association with these variables.

According to the findings of this study, the association between COC for diabetic complications and the probability of developing ESRD is consistent with the findings of previous studies, which emphasized that the better the COC, the better the outcome of various chronic conditions. Liao et al. revealed that diabetes mellitus patients with a high medical care-seeking consistency with a physician had a lower risk of diabetic complications compared with patients having a medium or low medical care-seeking consistency [35]. Christakis et al. further emphasized that children with a medium or high COC were less likely to be hospitalized for diabetic ketoacidosis [36]. Another study revealed that consistency in diabetic care increases patient satisfaction and decreases the risk of other chronic diseases [37]. The benefits of COC are enhanced among chronic disease patients because they tend to demand treatment more consistently than others without chronic diseases, who tend to engage in passive medical services utilization. Thus, the physician-patient relationship might be improved. Furthermore, improved COC was associated with a reduction in all-cause mortality, and prevented hospitalizations as reported in previous studies [38, 39].

The findings of our study indicate that improved COC among patients with nephropathy is associated with a lower probability of ESRD. Based on the patients’ socioeconomic statuses, some distinctions were observed among the variables. Specifically, with patients who lived in rural areas, the COC level was lower than that of those living in urban areas. In addition, based on the hospital characteristics, patients tend to visit clinics rather than general hospitals or hospitals, and so the COC index was higher among clinic users. The gap in the COC level influenced the probability of ESRD. According to our findings, patients who used medical services consistently had lower probability of ESRD than patients with lower COC index. This is consistent with the findings of previous studies, which emphasized on the importance of continuous diabetic care to prevent future hospitalizations, mortality, and excessive medical expenses. Furthermore, patients who used clinics for the care of diabetes had a lower probability of ESRD than patients who visited general hospitals [40, 41].

Among other characteristics, including the lowest income, and elderly patients, higher probability of ESRD was reported; in particular, patients with disabilities had much higher probability of ESRD than those without disabilities. However, there was no statistically significant difference in the COC among patients with disabilities.

As shown in the subgroup analysis, patients who lived in rural areas with worse COC had higher probability of ESRD. Although there was no difference by location in the subgroup analysis, in both regions the continuity of diabetic care among nephropathy patients was important to prevent future ESRD. Additionally, except for mid-high income level (Q3), improved COC level affected the probability of ESRD, and although this was not statistically significant, patients with bad COC levels had higher probability of ESRD. A similar trend was also observed for patients with disabilities and other covariates. With disability status, the proportion of ESRD was higher in patients with disabilities and those with the lowest income level. Although the socioeconomic status showed no direct effect on the probability of ESRD, and there was no significant gap in the consistency of diabetic care, there might be underlying disparities in the care of patients who are socially vulnerable, such as patients with disabilities or those in the lowest income level. Hence, these subgroups of patients should be aware of how to prevent the occurrence of ESRD. This is similar to the explanations from previous studies, which emphasized that poorer access to health care among black patients might explain their excess risk of ESRD, beyond the excess risk explained by demographic, socioeconomic, lifestyle, and clinical factors [42–45]. However, our study demonstrated the effect of COC on the probability of ESRD, and showed that patients with nephropathy (patients with renal complication) with bad COC level had higher probability of ESRD. In future research, the effect of COC on the actual change in clinical parameters, such as glycemic control, should be implemented to emphasize the importance of continuous care. Moreover, policy development to encourage COC among patients with diabetic complications, aimed at preventing severe chronic disease and managing diabetic complications more effectively in the future, should be implemented. There is need for further research aimed at minimizing the probability of ESRD among vulnerable classes of patients, among whom access to care is more difficult [46].

Our study has some limitations. First, we only used the procedure codes and ICD-10 codes from NHI claim data for defining ESRD. The procedures included hemodialysis, peritoneal dialysis, and kidney transplant. However, ESRD could be divided into several stages based on the disease severity while other clinical procedures could be used for defining ESRD. Hence, we might have underestimated the number of ESRD patients in our study. However, we defined ESRD by ICD-10 codes that were implemented for improving kidney functions; hence, the findings of our study showed that better consistency in diabetic nephropathy care resulted in a lower probability of ESRD.

Second, since we conducted a retrospective cohort study, with the exposure categorized as a dichotomous variable, misclassification was inherent. In cohort studies, exposure misclassification commonly occurs. If the assessment of exposure is implemented independently of the disease diagnosis, misclassification tends to result in spurious conclusion. Furthermore, not only with the exposure but also with certain covariates, using administrative data usually leads to misclassifications. Minimizing misclassification in future research should be ensured.

The third limitation is the lack of information on health behaviors such as perceived health status, smoking, depression, and drinking status, which might affect the incidence of ESRD. Health behaviors and blood glucose control are highly correlated, as revealed by findings of previous studies. Due to unmeasured or unknown factors, which might act as effect modifiers for the outcome (ESRD incidence), residual confounding might exist in this study, and this might have resulted in an imperfect adjustment, or could have led to spurious conclusions. To minimize this limitation, propensity score methods for determining the appropriate covariates to adjust for in the analysis should be applied in future research.

Moreover, we did not include hospital or doctor characteristics that might have had an influence on the probability of ESRD. In addition, we could not explain how the severity of diabetic renal complications affected the probability of ESRD, and with the limited data, we were unable to investigate whether study patients used outpatient prescriptions that might also have had an effect on the progression of ESRD. Furthermore, since the cause of death was not included in the health insurance qualification database, we could not clarify whether patients died as a result of diabetic complication or not. Since our focus was on the association between continuity of ambulatory care and the incidence of ESRD during the 8-year follow-up, we limited the analysis to survivals during follow-up period. Therefore, the number of patients might have been underestimated in this study.

The fourth limitation is that we did not evaluate the COC in multiple ways. We focused on the COC based on the COC index alone. However, there are several other indices such as the Usual Provider Care index and the Sequential Continuity of Care index; hence, we were unable to explain which COC index might explain the probability of ESRD better. Moreover, despite our inclusion of the patients’ individual- and hospital-level characteristics as covariates in the main analysis, we failed to test for correlations within hospitals. Since we only determined the association of COC among diabetic nephropathy patients, further research should be implemented to also assess the correlation within hospitals among the care episodes.

Furthermore, because of inappropriate handling of missing data, there is a potential of the effect estimate (HR) to be biased towards the null. Since we determined the association between COC and the incidence of ESRD among survivors during the 8-year follow-up, the exclusion of patients who died tends to underestimate the number of diabetic nephropathy patients. Furthermore, we excluded inpatients or health center users. To assess the effect of the COC on the incidence of ESRD, all outpatient medical use history should be included. To minimize potential selection bias, more refined sampling methods are needed, such as matching, or including the appropriate control group.

Despite these limitations, our study has several strengths. First, we analyzed a representative sample of patients with diabetic renal complications using nationwide claims data in Korea. Secondly, unlike most previous studies, we included each patient’s socioeconomic status, which could define the difference in COC between socially vulnerable patients, including patients with lower income level, or those with or without disabilities.

Our results support the hypothesis that reducing fragmented care and improving COC among diabetic complication patients can decrease the probability of ESRD. We therefore encourage policymakers to recognize the need for an effective healthcare delivery system that promotes COC. Furthermore, to enhance accessibility, we suggest the need to undertake studies aimed at examining the relationship between medical service providers and patients with diabetic complications. In addition, to prevent the progression of ESRD among patients with type 2 diabetes mellitus with renal complications, it is necessary to undertake measures aimed at improving the sustainability of patients with disabilities. Continuous efforts of medical staff and changes in the national policy for chronic disease management are necessary to improve outpatient care for the sustainable use by patients.

## Conclusion

In conclusion, we measured COC among newly diagnosed nephropathy patients during the entire study period and analyzed the relationship between the COC and the probability of ESRD. According to our results, higher continuity of care was associated with lower risk of ESRD among patients with diabetic renal complication. To prevent ESRD among diabetic renal complication patients, continuous follow-up should be encouraged. Moreover, there were disparities in health outcomes between socially vulnerable groups. To bridge the gap in health outcomes among the different socio-economic patient groups, accessibility of care without disparities should be guaranteed. Further research is needed on the associations between long-term COC and various healthcare outcomes, including medical expenses or patient’s quality of life. Moreover, to ensure that the study findings could be generalized to the larger population of patients, long-term follow-up of patients with diabetic complications would be needed. Health behavior, changes in the socioeconomic status, and disease progression mechanisms, which might mediate the utilization of medical services and health outcomes, should be considered.

## Additional file

Additional file 1: **Table S1.** Sensitivity analysis of the association between continuity of care and ESRD incidence (including deaths). (DOC 31 kb)

## Abbreviations

CI: Confidence interval
COC: Continuity of care
ESRD: End-stage renal disease
HR: Hazard ratio
ICD-10: International Classification of Diseases tenth edition diagnosis code
KDRG: Korean Diagnosis-Related Group
NHI: National Health Insurance
PCCL: Patient Clinical Complexity Level
Q: Quartile
SD: Standard deviation
