# Supplementary material for: The effect of continuity of care on the incidence of end-stage renal disease in patients with newly detected type 2 diabetic nephropathy: a retrospective cohort study
Source: BMC Nephrol. 2018 Jun 5;19:127. doi: 10.1186/s12882-018-0932-3 (PMC5989468; doi:10.1186/s12882-018-0932-3)
Supplement: Supplementary file 1 — Table S1. Sensitivity analysis of the association between continuity of care and ESRD incidence (including deaths). (DOC 31 kb) [file 12882_2018_932_MOESM1_ESM.doc]

| **Table S1. Sensitivity analysis of the association between continuity of care and ESRD incidence (including deaths)** | | | | | | |
| --- | --- | --- | --- | --- | --- | --- |
|  | | **ESRD incidence** | | | |  |
|  |  | **Hazard Ratio** | **95% CI** | | |  |
| **Continuity of care** | |  |  |  |  |  |
|  | Good (COC index≥0.75) | 1.00 | - |  | - |  |
|  | Bad (COC index<0.75) | 1.95 | (1.24 | - | 3.07) |  |
| *Adjusted for sex, age, residence area, health insurance type, disability type, insulin treatment, PCCL index, hospital classification, number of beds, number of doctors, and hospital location | | | | | | |
